# Cryptic variation in an ecological indicator organism: mitochondrial and nuclear DNA sequence data confirm distinct lineages of *Baetis harrisoni* Barnard (Ephemeroptera: Baetidae) in southern Africa

**DOI:** 10.1186/1471-2148-12-26

**Published:** 2012-02-29

**Authors:** Lyndall L Pereira-da-Conceicoa, Helen M Barber-James, Nigel P Barker, Ferdy C de Moor, Martin H Villet

**Affiliations:** 1 Department of Zoology and Entomology, Rhodes University, P.O. Box 94, Grahamstown, 6140, South Africa; 2 Department of Freshwater Invertebrates, Albany Museum, Somerset Street, Grahamstown, 6140, South Africa; 3 Department of Ecology & Evolutionary Biology, University of Connecticut, Storrs, 06269, CT, USA; 4 Molecular Ecology & Systematics Group, Department of Botany, Rhodes University, P.O. Box 94, Grahamstown, 6140, South Africa

## Abstract

**Background:**

*Baetis harrisoni* Barnard is a mayfly frequently encountered in river studies across Africa, but the external morphological features used for identifying nymphs have been observed to vary subtly between different geographic locations. It has been associated with a wide range of ecological conditions, including pH extremes of pH 2.9–10.0 in polluted waters. We present a molecular study of the genetic variation within *B. harrisoni* across 21 rivers in its distribution range in southern Africa.

**Results:**

Four gene regions were examined, two mitochondrial (*cytochrome c oxidase subunit I* [*COI*] and *small subunit ribosomal 16S rDNA* [*16S*]) and two nuclear (*elongation factor 1 alpha* [*EF1α*] and *phosphoenolpyruvate carboxykinase* [*PEPCK*]). Bayesian and parsimony approaches to phylogeny reconstruction resulted in five well-supported major lineages, which were confirmed using a general mixed Yule-coalescent (GMYC) model. Results from the *EF1α* gene were significantly incongruent with both mitochondrial and nuclear (*PEPCK*) results, possibly due to incomplete lineage sorting of the *EF1α* gene. Mean between-clade distance estimated using the *COI* and *PEPCK* data was found to be an order of magnitude greater than the within-clade distance and comparable to that previously reported for other recognised *Baetis* species. Analysis of the Isolation by Distance (IBD) between all samples showed a small but significant effect of IBD. Within each lineage the contribution of IBD was minimal. Tentative dating analyses using an uncorrelated log-normal relaxed clock and two published estimates of *COI* mutation rates suggest that diversification within the group occurred throughout the Pliocene and mid-Miocene (~2.4–11.5 mya).

**Conclusions:**

The distinct lineages of *B*. *harrisoni* correspond to categorical environmental variation, with two lineages comprising samples from streams that flow through acidic Table Mountain Sandstone and three lineages with samples from neutral-to-alkaline streams found within eastern South Africa, Malawi and Zambia. The results of this study suggest that *B*. *harrisoni* as it is currently recognised is not a single species with a wide geographic range and pH-tolerance, but may comprise up to five species under the phylogenetic species concept, each with limited pH-tolerances, and that the *B*. *harrisoni* species group is thus in need of taxonomic review.

## Background

It is axiomatic that successful applied biology relies on sound taxonomy. For instance, the success of biomonitoring depends in part on the correct identification of the indicator organisms involved [1]. Mayflies are known to be important indicator species in aquatic biomonitoring (e.g. [2-5]). Amongst these, two species of mayflies in the genus *Baetis* Leach stand out in importance: *B*. *rhodani* Pictet in Europe (e.g. [6-8]), and *B*. *harrisoni* Barnard in southern Africa (e.g. [2,9-11]). *Baetis rhodani* has long been recognised as a complex of cryptic species (see [12] for a review). The research on *B. harrisoni* presented here investigates whether this species may be similar to *B. rhodani* in terms of its widespread distribution and range of ecological tolerances, and whether it also represents a number of cryptic species.

*Baetis harrisoni* is a physically robust species, with nymphs found in slow- to fast-flowing (0.1–1.0 ms^−1^) streams and rivers throughout sub-Saharan Africa [13]. It has also been recorded in polluted waters ranging in pH from about 2.9 to 10.0 [9,14]. Such a broad geographical distribution and variation in environmental tolerance in an organism with dubious dispersal ability suggests that there may be cryptic species associated with the name. A third line of evidence that *B*. *harrisoni* may constitute more than one species is that the morphological features used to identify its nymphs (e.g. mouthpart structure, abdominal pigmentation and relative size of gills) have been observed to vary subtly between populations in different geographic locations. *Baetis harrisoni* is therefore worth studying as a species of practical significance but uncertain taxonomy.

The use of molecular (DNA) data in resolving cryptic lineages has at least four advantages. Firstly, DNA data facilitate the detection of cryptic species because they provide more direct evidence of population genetic processes like interbreeding than do morphological data, and often with greater precision. For example, recent studies using the mitochondrial *cytochrome c oxidase I* (*COI*) gene showed that the name *B*. *rhodani* was applied to at least nine morphologically cryptic haplogroups [15] and that in Finland the species *B*. *macani* Kimmins and *B*. *vernus* Curtis are both paraphyletic in terms of their *COI* gene phylogenies [16]. Secondly, molecular markers provide a valuable benchmark against which morphological characters for each lineage can be sought, and facilitate the interpretation of morphological variability. A re-analysis of morphological characters of the nymphs of *B*. *macani* (following a molecular study) provided diagnostic markers for identification of some lineages identified by DNA haplotypes [16]. Thirdly, DNA data facilitate the association of conspecific immature and adult stages in life-cycles that feature radical metamorphosis and this has been done successfully in some mayflies [17]. Finally, by helping to resolve questions of relationship amongst specimens from different places with greater precision, molecular markers can provide information about historical and geographical processes that underlie diversity.

Mitochondrial DNA sequences (especially of the *COI* region) provide useful data for addressing the types of challenges raised here [15,16,18]. However, numerous critiques of the taxonomic use of this gene [19-23] emphasise the need to cross-validate phylogenies with data from additional nuclear genes. This need arises from the possibility of incomplete lineage sorting, horizontal gene transfer, introgression and population fragmentation effects [24]. For example, two specimens identified by their morphology as members of *B*. *macani* and *B*. *liebenauae* Keffermüller, respectively, were identified as *B*. *vernus* using *COI*-based phylogenetic analysis, suggesting mitochondrial introgression [16]. Previous molecular taxonomic studies of *Baetis* species [15,16] have used only mitochondrial sequence data.

Our study has two aims. First, it provides a molecular study of the genetic variation within *B*. *harrisoni* across its distribution range in southern Africa using four genes (*COI*, *16S*, *PEPCK* and *EF1α*). Second, it explores two nuclear genes (*PEPCK* and *EF1α*) as additional nuclear DNA markers to cross-validate the results of *COI* data, given recent critiques of using this region in isolation.

## Methods

### Taxon sampling

**Figure 1:**
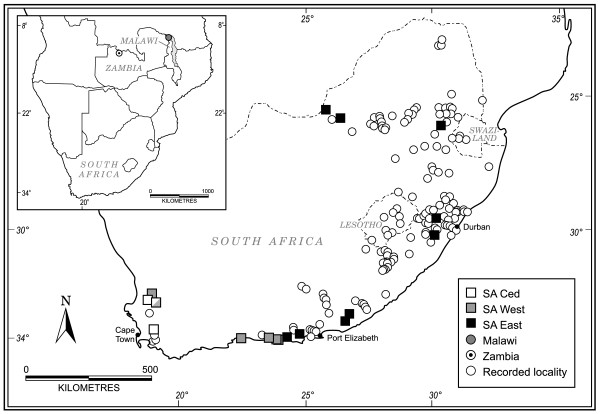
**Map of recorded and sampled localities.** Recorded localities of *B. harrisoni* and the location of sampled sites corresponding to the major lineages of *B*. *harrisoni* in Southern Africa. Data obtained from the Albany Museum database.

Our sampling strategy was driven by seeking cryptic variation where one is most likely to find it. *Baetis harrisoni* occurs throughout the south and east of South Africa (Albany Museum records: Figure 1), in biomes and climates that vary from Mediterranean (with winter rainfall) in the southwest Cape Floristic Region to temperate (with aseasonal rainfall) in the south-eastern Albany Thicket, to subtropical (with summer rainfall) in the north-eastern Savanna. In addition the western and southern Cape rivers are acidic, and those of the east neutral to alkaline [25]. This environmental heterogeneity is probably greater than would be found in the species’ distribution in the rest of Africa, and if *B*. *harrisoni* did contain independent cryptic taxa, they are most likely to occur here. To contextualise the rest of Africa, specimens of *B*. *harrisoni* from Zambia and Malawi were included in the study. 

At least ten nymphs of *B*. *harrisoni* were collected from each of 19 rivers spread across South Africa and one river in each of Zambia and Malawi (Figure 1, see Additional file 1: Table S1) and preserved in 96% ethanol. Two or three specimens from each river were used for molecular analysis, while the remaining specimens were kept for future morphological study. Nymphs of *B*. *rhodani* were provided by Dr Jean-Luc Gattolliat (Museum of Zoology, Switzerland) to serve as an outgroup. Vouchers of samples extracted for DNA (heads and the first pair of legs, or whole exoskeletons) are housed at the Albany Museum, Grahamstown (AMGS).

### Molecular data

DNA was extracted either from the entire abdomen using the Chelex® 100 extraction protocol [26] or by internal body digestion using the Invisorb extraction kit (Invitek).

Partial segments of four gene regions were amplified and sequenced, two mitochondrial (*cytochrome c oxidase subunit ICOI* and *small subunit ribosomal 16S rDNA16S*) and two nuclear (*elongation factor 1 alphaEF1α* and *phosphoenol pyruvate carboxykinasePEPCK*). The primers used for both the PCR and sequencing reactions were as follows: *COI* was amplified using C1-N-2568 [27] and C1-J-1718 [28]; *16S* was amplified using *16S*ar and *16S*br [29]; *EF1α* was amplified using DV-EF-F1 (5′- CAGGAYGTATACAAAATTGGTGG -3′) (Vanderpool, unpublished) and DALL (5′- CTACACACATTGGTTTGCTGGG -3′) designed for this study; *PEPCK* was amplified using pepFb12 (5′- GGAACTTCAAACAGCACCAAT -3′) and pepRb45 (5′- ACCTTGTGTTCTGCAGCT -3′) (modified by Vuataz, from [30]). All PCR reactions were performed in a 50 μl volume using the following thermal cycling profile: initial denaturation of 94°C for 5 min, followed by 35–40 cycles of denaturation at 94°C for 30s, primer annealing at 48°C (*COI* and *16S*), 53°C (*EF1α*) or 52°C (*PEPCK*) for 30s, elongation at 72°C for 1 min 30s; followed by a final extension period of 72°C for 10 min. PCR products were cleaned using the Wizard® SV (Promega Corp.) and Invisorb PCRapace (Invitek) quick purification kits.

Cycle sequencing of the cleaned PCR product was carried out in both directions for each gene region using the flanking PCR primers and the ABI Big Dye Sequencing kit v.3.1, according to the manufacturer’s instructions. Sequence trace files were generated using an ABI 3100 genetic analyser situated at Rhodes University. Trace files were checked and edited using GeneStudio v.2 (GeneStudio, Inc). Sequences were initially aligned using the ClustalW algorithm in MEGA v.4 [31] using the default parameters and then refined manually. Gaps in the *16S* alignment were treated as missing data and there were no introns in the *PEPCK* or *EF1α* alignments. Sequences are available through GenBank [GenBank: HM636930-HM637101] (see Additional file 1: Table S1).

### Phylogenetic analyses

To test whether the data from each dataset (corresponding to each gene region) could be combined into a single data set, pairwise Incongruence Length Difference (partition homogeneity) tests [32] were conducted in PAUP* v.4.0b10 [33], using 1000 replicates. Each gene was tested for substitution saturation using plots of transitions and transversions against F84 distance in DAMBE v4.5 [34]. Analyses were then conducted using Maximum Parsimony (MP) and Bayesian Inference (BI) for each dataset independently and the combined molecular data.

Each gene dataset was tested for the most appropriate model of sequence evolution using the AIC test [35] as implemented in MrModeltest v.2.2 [36] using MrMTgui (available from http://genedrift.org/software/mrmtgui.html).

Bayesian Inference analyses were conducted using MrBayes v.3.1.2 [37] for each of the datasets. Each BI analysis comprised two independent runs each of ten million generations. Random starting trees with four chains (one cold, three hot) were used with trees sampled every 1000 generations. All model parameters except branch length and topology were unlinked across partitions and among-partition rate variation was accommodated following Marshall et al. [38]. Stationarity was assessed using the Potential Scale Reduction Factor data and plots of likelihood scores, tree length and average standard deviation of split frequencies against the number of generations. The first 1000 trees sampled (10%) were discarded from each run as burn-in. The majority rule consensus Bayesian topology and posterior probability values were then computed from the remaining sampled trees. For the combined analysis the effect of partitioning the data by gene was estimated using pairwise comparison of Bayes Factors (*sensu*) [39,40] with 2lnBF_A–B_ ≥ 10 indicative of strong support for partitioning strategy A vs. B, following Kass and Raftery [41]. Although popular, the harmonic mean (HM) estimate of the marginal likelihood as reported by MrBayes is a biased estimator of the marginal likelihood [42] with a large and unpredictable variance [43]. These properties render the HM inappropriate for Bayes Factor comparisons, instead the more recent stepping stone (SS) method is recommended [42,44]. Following analysis in MrBayes the marginal likelihood of the trees resulting from the two partitioning strategies were re-estimated using the generalized stepping stone (SS) method [44] as implemented in Phycas 1.2.0 http://www.phycas.org using 30 β-values with 1000 cycles per β.

Parsimony analyses were performed using the heuristic search option in PAUP* version 4.0b10 [33]. A simple search with TBR (Tree Bisection and Reconnection) branch-swapping was used to find the approximate length of the shortest trees. This was followed by a random input analysis using 1000 repetitions and TBR branch-swapping, keeping all trees equal to or shorter than the shortest tree found in the simple search. This process was repeated until no shorter trees were found. All of the shortest trees were retained and used to compute the strict consensus tree. Nodal support was investigated using 100 bootstrap pseudoreplicates [45] with MAXTREES set to 10 000, TBR branch-swapping and simple stepwise addition.

To estimate the contribution of each dataset to the overall support of each node, partitioned Bremer support (PBS) values [46-49] were calculated using TreeRot v.3 [50] and PAUP* using only the parsimony informative characters for each dataset and 1000 random addition replicates. As only the major lineages were of interest, the PBS analysis used a reduced dataset comprising only individuals from each clade represented with the complete molecular dataset.

Both mean within- and between-clade distances were estimated from the *COI* and *PEPCK* data using the Maximum Composite Likelihood model in MEGA 4 [31]. In addition, the contribution of Isolation by Distance (IBD) was estimated using the most informative gene data set (*COI*) in GenAlEx 6.1 [51] with 999 permutations using the Mantel test in GenAlEx 6.1, initially for the group as a whole and subsequently for each clade individually, excluding the Zambian and Malawian clades that originated from one site each, preventing analysis.

### Dating analysis

As no fossils were available, a tentative dating analysis used minimum (1.5% per MY; [52]) and maximum (3.5% per MY; [53]) reported values for *COI* substitution rates of insects calibrated within the last 10 MY. Divergence times were estimated using BEAST v1.6.1 [54] as follows. Data were partitioned by gene (*PEPCK*, *16S*, *COI*) and the model parameters selected by MrModeltest for each partition (Table 1) were incorporated into each analysis. Each analysis comprised four independent runs of 50 million generations, with a UPGMA (Unweighted Pair Group Method with Arithmetic Mean) starting tree under the assumptions of an uncorrelated log-normal (UCLN) relaxed clock. The *PEPCK* and *16S* substitution rates were estimated relative to the *COI* rate (*COI* rate set at 0.0075 or 0.0177 substitutions per site per MY) in BEAST. Each analysis was run under either a Coalescent (Constant Size), Yule, or Birth-Death prior to ascertain the affect of these tree priors on the resulting divergence estimates. Run statistics including the effective sample size for each parameter were examined in Tracer v1.5 [55] and convergent independent runs for each analyses were then combined in Log Combiner 1.6.1 (part of the BEAST package) with a final burn-in (10%) of the trees. Node ages and the corresponding confidence intervals were then summarised using a Maximum Clade Credibility (MCC) tree in TreeAnnotator v1.6.1 (part of the BEAST package). These trees and corresponding node ages were viewed using FigTree v1.3.1 (part of the BEAST package). 

**Table 1:** Data characteristics and analysis summaries

		**Characters**				**Parsimony analysis**				**Bayesian analysis**	
**Dataset**	**ntax**	**bp**	**# Var**	**# Pi**	**% Pi**	**# trees**	**Score**	**CI**	**RI**	**Model (AIC)**	**lnL**
*PEPCK*	52	357	58	33	9.2	10000	49	0.776	0.969	K80 + G	−1063.23 (1P)
*EF1α*	21	458	33	14	3.1	5	18	0.833	0.957	GTR + I	−913.51 (1P)
*16S*	50	502	71	65	13.0	10000	104	0.779	0.965	GTR + I	−1393.33 (1P)
*COI*	50	618	224	191	31.0	15	424	0.642	0.936	GTR + I + G	−3016.22 (1P)
*PEPCK + 16S + COI*	67	1477	353	289	20.0	10000	586	0.667	0.943	GTR + I + G	−5724.33* (1P) −5315.31* (3P)

The number of specimens with sequence data (ntax), number of variable (# Var), parsimony informative (# Pi), percent parsimony informative (% Pi) and total number of base pairs (bp) is reported. The parsimony search summary comprises the number of trees retained (# trees), tree length (score), Consistence Index (CI) and Retention Index (RI). The Bayesian analysis summary comprises the model selected by MrModeltest, the harmonic mean of the estimated marginal likelihoods and the partitioning strategy indicated in brackets; an asterisk indicates the value is the re-estimated marginal likelihood using the generalized stepping stone model.

### Species delimitation

Species boundaries were explored using changes in branching rates following Pons et al [56]. Both single and multiple thresholds, general mixed Yule-coalescent (GMYC) models were applied to each gene in isolation and to the combined data. After removing the outgroup specimens and all duplicate haplotypes, each chronogram was estimated using BEAST under the appropriate gene-specific model parameters (Table 1), with a relaxed uncorrelated log-normal (UCLN) clock, the mean substitution rate fixed at 1 and branch lengths estimated using a Coalescent (Constant Size) prior. Analyses were run for 50 million generations. Following the BEAST analyses the Maximum Clade Credibility chronograms were analysed using the SPLITS (available from http://r-forge.r-project.org/projects/splits) and APE [57] packages in the R statistical environment (R Development Core Team, 2011). A log-likelihood ratio test was then used to assess the significance of the estimated shift in branching rates for both the single and multiple threshold models in each analysis.

## Results

### Data characteristics

The combined molecular data consisted of 67 specimens and 1935 nucleotides (*16S* = 502 bp; *PEPCK* = 357 bp; *COI* = 618 bp; *EF1α* = 458 bp) obtained from specimens from 22 rivers (see Additional file 1: Table S1; Figure 1) including outgroups. Certain sample sequences were successfully amplified for some genes and not others and are therefore absent in certain gene analyses (see Additional file 1: Table S1). Of the four genes, *COI* showed the most variation and *EF1α* the least (Table 1). Individual plots of transitions and transversions against F84 distance for the ingroup samples showed no signs of saturated substitution within the four datasets (data not shown).

### Phylogenetic analyses

**Figure 2:**
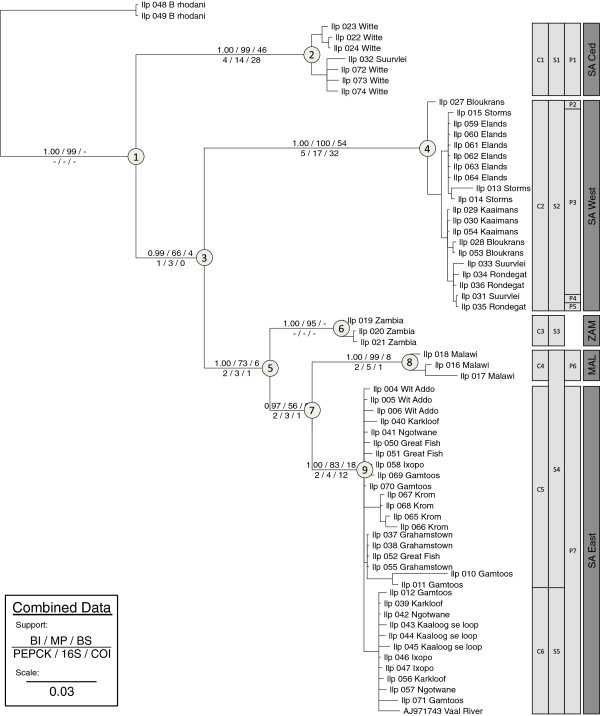
**Combined Bayesian Inference with GMYC.** Bayesian Inference phylogram of combined molecular data (excluding *EF1α* data) with support for major nodes shown above (Bayesian posterior probability / Parsimony bootstrap / Bremer support) and below (partitioned Bremer support: *PEPCK* / *16S* / *COI*) each branch. The columns to the right of the tree indicate species boundaries identified by the GMYC likelihood analyses for the *COI* (clades: C1–C6), *16S* (clades: S1–S5) and *PEPCK* (clades: P1–P7) partitions respectively. The fourth (dark grey) column shows five well supported monophyletic consensus groups identified by the GMYC likelihood analyses of the combined data (*PEPCK* + *16S* + *COI*), representing putative phylogenetic species further discussed in this study. Node numbers (circled) correspond to date estimates reported in Tables 3 &4 and Additional file 2: Figure S1.

The results of the Parsimony and Bayesian analyses are summarised in Table 1. Pairwise ILD tests showed that three of the four datasets were not significantly incongruent, with the exception of *EF1α* which was significantly incongruent from the other three genes (Table 2). Comparison of the two partitioning strategies in the combined Bayesian analyses showed no change in topology but an improvement in the support of a single node (Figure 2: the posterior probability of node 3 improved from 0.62 [single partition] to 0.97 [three partitions]). Comparison of the marginal likelihoods estimated using the generalized stepping stone (SS) method suggested that partitioning data by gene provided a much better ln-likelihood value (2lnBF_1–3_ = 818.04) than not partitioning the data for each gene (Table 1), and thus the results of the Bayesian analysis partitioning the data by gene (three partitions) are shown (Figure 2).

**Table 2:** Pairwise Incongruence Length Difference (ILD)

**Partition**	***EF1α***	***PEPCK***	***16S***
*EF1α*	-		
*PEPCK*	**0.01**	-	
*16S*	**0.01**	0.61	-
*COI*	**0.01**	0.20	0.74

ILD test probability values for the four data partitions generated using 1000 replicates. Statistically significant values are emphasised in bold.

Five clades were found, consisting of samples collected from the Cederberg mountains and the Breede River district, which is a winter rainfall region of South Africa hereafter termed “SA Ced”; the entire winter rainfall region of South Africa, Cederberg inclusive, hereafter termed “SA West”; and the eastern summer rainfall region of southern Africa, corresponding to samples from South Africa “SA East”; Malawi, “MAL”; and Zambia, “ZAM”. The arrangement of the various clades differed depending on the data analysed.

**Figure 3:**
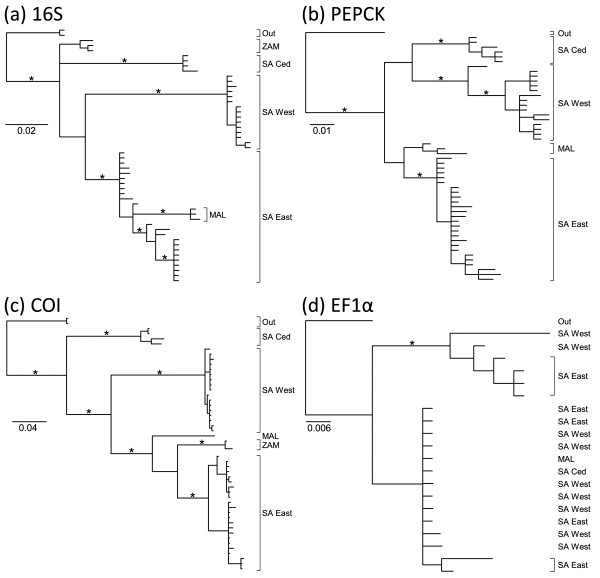
**Bayesian Inference Phylograms.** Bayesian Inference phylograms of each of the four different datasets corresponding to each individual gene: (**a**) *16S* (**b**) *PEPCK* (**c**) *COI* (**d**) *EF1α*. Posterior probability support (*p* > 0.95) is indicated using an asterisk above the corresponding branch.

Analysis of the *16S* data resulted in a basal polytomy (Figure 3a). Two clades (SA Ced and SA West) were recovered as monophyletic; samples from eastern rivers were recovered as two paraphyletic clades (SA East + MAL) and (ZAM). Three clades (SA Ced, SA West and (SA East + MAL)) received good support (MP: ≥ 82; BI: ≥ 0.94) while the clade from Zambia (ZAM) received moderate support (MP: 82; BI: 0.81). Analysis of the *PEPCK* data (Figure 3b) resulted in three clades (SA Ced, SA West and (SA East + MAL)), with two (SA Ced and SA West) receiving good support (MP: ≥ 90; BI: 1.00). Within the eastern lineage the SA East samples formed a well-supported clade (MP: ≥ 90; BI: ≥ 0.98), whereas the samples from Malawi (MAL) were monophyletic but only moderately supported (MP: 63; BI: 0.87) and the samples from Zambia (ZAM) did not amplify for this gene. Analysis of the *COI* data (Figure 3c) indicated five clades (SA Ced, SA West, SA East, MAL and ZAM), each with good support (MP: ≥ 99; BI: 1.00). The trees recovered from analyses of the *EF1α* data (Figure 3d) were poorly supported and incongruent with the trees produced from the other genes, both when analysed separately (Figure 3a-c) and when combined in a total evidence analysis (Figure 2). Specimens that were represented in distinct geographic clades within the *16S*, *PEPCK* and *COI* analyses were indistinguishable in the *EF1α* analysis (Figure 3d).

Considering the limited phylogenetic information provided by the *EF1α* data (Table 1) and their significant incongruence with all other sampled datasets (Table 2), the *EF1α* gene was excluded from the combined analysis (Figure 2). The MP and BI analyses of the combined remaining molecular data (comprising *16S*, *PEPCK* and *COI* datasets) recovered five monophyletic lineages (SA Ced, SA West, SA East, MAL and ZAM). Three lineages (SA Ced, SA West and a combined “eastern” clade comprising (SA East + MAL + ZAM)) received good support (MP: ≥ 73; BI: 1.00). Within the eastern clade the Zambian (ZAM) lineage was recovered as sister to the combined Malawian (MAL) and South African (SA East) lineages, which received moderate support (MP: 56; BI: 0.97). In addition the sister relationship of ((SA East + MAL + ZAM) and SA West) received moderate support (MP: 66; BI: 0.99). The PBS analysis indicated little conflict between the three (*16S*, *PEPCK* and *COI*) datasets, with very good support for each clade (combined PBS ≥ 8) but only moderate support for the between-clade relationships (combined PBS ≤ 6).

The within-clade distance estimated using the Maximum Composite Likelihood model was comparatively low (*COI*: mean = 1.8%, range = 0.5–3.0%; *PEPCK*: mean = 0.7%, range = 0.2–0.9%), whereas the between-clade distance was an order of magnitude greater (*COI*: mean = 17.6%, range = 11.6–22.5%; *PEPCK*: mean = 4.4%, range = 2.4–5.7%) and comparable to the distance between the ingroup and outgroup taxa (*COI*: mean = 22.4%; range = 21.1–23.6%; *PEPCK*: mean = 7.5%, range = 6.7–9.6%).

Isolation by Distance (IBD) analyses of the samples as a whole showed a small but significant effect of IBD (R^2^ = 0.084; *p* = 0.001). The contribution of IBD was minimal within each individual clade (Table 3).

### Dating analysis

The topology of the various BEAST dating analyses (data not shown) agreed in general with the MrBayes analysis (Figure 2), with two exceptions: the analysis under a Yule prior and a *COI* substitution rate of 1.5% per MY (Table 4) and the analysis under a Birth-Death prior and a *COI* substitution rate of 3.5% per MY (Table 5), both of which did not recover a monophyletic clade comprising (SA East + MAL + ZAM + SA West: Figure 2, node 3) due to a weakly supported clade comprising (SA West + SA Ced). Unsurprisingly divergence estimates were affected by both the tree prior and the substitution rate used (Tables 3 &4; Additional file 2: Figure S1). The fast substitution rate (3.5% per MY) resulted in divergence estimates which were approximately half those estimated using the slow substitution rate (1.5% per MY) across the whole range of estimates. The effect of tree prior was relatively minor for the within clade estimates (Tables 3 &4; Additional file 2: Figure S1) but the between clade divergence estimates were strongly affected by the tree prior with a Yule prior favouring divergence estimates that were approximately half of those estimated using the Coalescent and Birth-Death priors (Tables 3 &4; Additional file 2: Figure S1). Comparisons of the individual BEAST runs using the Bayes Factor calculator in Tracer with 1000 bootstrap replicates showed that a Yule prior (3.5% Ln = −5294.406; 1.5% Ln = −5295.392) was favoured over both the Coalescent prior (3.5% Ln = −5295.698; 1.5% Ln = −5296.326) and Birth-Death prior (3.5% Ln = −5296.363; 1.5% Ln = −5295.95), although this support was only marginal (2lnBF_A-B_ ≤ 4 in all pairwise comparisons).

Within each of the lineages the mean time to most recent common ancestor (TMRCA) ranged from the youngest: 0.5–0.7 mya (MAL) to the oldest: 1.5–1.7 mya (SA East) using the faster rate (Table 5) or 1.1–1.6 mya (MAL) to 3.4–3.9 (SA East) using the slower rate (Table 4). The ingroup taxa are estimated to share a common ancestor in the mid- to late Miocene at least 5.2 mya (fast rate, Yule prior)–24.6 mya (slow rate, Birth-Death prior). The most recent cladogenesis between major lineages occurred at least 2.4 mya (fast rate, Yule prior)–8.9 mya (slow rate, Birth-Death prior) between the SA East and MAL lineages (Table 4).

**Table 3:** Isolation by Distance (IBD)

**Clade**	**R**^**2**^	***P***
INGROUP	0.084	**0.001**
SA Ced	0.338	0.077
SA West	0.064	**0.022**
SA East	0.132	**0.001**
SA East + MAL + ZAM	0.717	**0.001**

The contribution of Isolation by Distance (R^2^) and corresponding probability values (*p*) for each clade analysed using the *COI* gene. Statistically significant values are emphasised in bold.

**Table 4:** TMRCA with substitution rate at 1.5% per My

		**Coalescent prior**		**Yule prior**		**Birth-Death prior**	
**Clade**	**Node**	**TMCRA (mya)**	**95% HPD**	**TMCRA (mya)**	**95% HPD**	**TMCRA (mya)**	**95% HPD**
MAL	8	1.27	(0.19–2.86)	1.63	(0.29–3.36)	1.14	(0.15–2.58)
ZAM	6	1.55	(0.19–3.63)	1.88	(0.24–4.16)	1.45	(0.19–3.38)
SA Ced	2	2.19	(0.65–4.23)	2.11	(0.65–3.94)	1.99	(0.60–3.79)
SA West	4	3.04	(1.04–5.65)	3.54	(1.35–6.19)	2.71	(0.93–5.12)
SA East	9	3.92	(1.97–6.30)	3.45	(1.66–5.73)	3.84	(1.80–6.29)
(SA East + MAL)	7	8.19	(3.91–13.09)	5.75	(2.81–9.60)	8.85	(3.92–14.72)
(SA East + MAL + ZAM)	5	12.74	(5.96–20.13)	7.74	(3.68–12.71)	13.92	(6.30–22.81)
(SA East + MAL + ZAM + SA West)	3	18.80	(9.41–29.73)	-	-	20.09	(9.41–32.88)
(SA East + MAL + ZAM + SA West + SA Ced)	1	24.23	(12.68–38.46)	11.49	(5.64–18.63)	24.63	(11.41–39.94)

Mean and 95% HPD estimates of the time to most recent common ancestor (TMRCA) estimated using a *COI* substitution rate of 1.5% per My in BEAST. Node numbers correspond to those in Figure 2; “-” represents node present in Figure 2 but absent in BEAST analysis. Clades shown in ascending order of age of MRCA.

**Table 5:** TMRCA with substitution rate at 3.5% per My

		**Coalescent prior**		**Yule prior**		**Birth-Death prior**	
**Clade**	**Node**	**TMCRA (mya)**	**95% HPD**	**TMCRA (mya)**	**95% HPD**	**TMCRA (mya)**	**95% HPD**
MAL	8	0.59	(0.08–1.30)	0.70	(0.13–1.55)	0.54	(0.09–1.20)
ZAM	6	0.71	(0.08–1.68)	0.85	(0.10–1.92)	0.64	(0.06–1.52)
SA Ced	2	0.96	(0.25–1.87)	0.94	(0.28–1.77)	0.89	(0.26–1.76)
SA West	4	1.41	(0.48–2.64)	1.60	(0.62–2.86)	1.29	(0.46–2.41)
SA East	9	1.73	(0.80–2.92)	1.54	(0.72–2.59)	1.71	(0.81–2.83)
(SA East + MAL)	7	3.64	(1.71–6.08)	2.40	(1.25–4.29)	3.96	(1.69–6.65)
(SA East + MAL + ZAM)	5	5.67	(2.50–9.48)	3.46	(1.66–5.75)	6.24	(2.71–10.52)
(SA East + MAL + ZAM + SA West)	3	8.16	(3.85–13.51)	4.33	(2.20–6.95)	-	-
(SA East + MAL + ZAM + SA West + SA Ced)	1	10.42	(4.90–17.33)	5.15	(2.66–8.51)	13.23	(5.45–22.47)

Mean and 95% HPD estimates of the time to most recent common ancestor (TMRCA) estimated using a *COI* substitution rate of 3.5% per My in BEAST. Node numbers correspond to those in Figure 2; “-” represents node present in Figure 2 but absent in BEAST analysis. Clades shown in ascending order of age of MRCA.

### Species delimitation

The results of the individual GMYC analyses are summarised in Table 6. In all cases the multiple-threshold model was not significantly different from the single model and was rejected in favour of the more conservative single threshold model. The *COI* data recovered six ML entities (Figure 2, C1–C6) the *16S* data recovered five ML entities (Figure 2, S1–S5) and the *PEPCK* data recovered seven ML entities (Figure 2, P1–P6), but none of these outcomes were significantly different from the null model of a single species. However, the combined *COI* + *16S* + *PEPCK* data recovered six ML entities, which was significantly different from the null model of a single species (*p* = 0.04). Five of these entities correspond to monophyletic, well supported clades (Figure 2).

**Table 6:** Summary of GMYC

			**Single threshold**					**Multiple threshold**				
**Dataset**	**H/G**	**Ln (null)**	**Ln (GMYC)**	**LR**	***p***	**ML entities**	**CI**	**Ln (GMYC)**	**LR**	***p***	**ML entities**	**CI**
*COI*	48	161.10	163.11	4.03	0.25	6	4–18	163.72	1.50	0.68	9	5–13
*16S*	48	106.58	108.46	3.75	0.29	5	1–15	163.58	0.00	1.00	5	1–11
*PEPCK*	54	166.48	169.30	5.62	0.13	7	4–12	169.49	0.38	0.94	6	4–15
*PEPCK + 16S + COI*	64	169.65	173.60	7.90	**0.04**	6	4–17	173.84	0.48	0.92	7	6–32

Summary of the single and multiple threshold GMYC analyses, including the number of unique haplotypes/genotypes (H/G) in each dataset; the likelihood values estimated under the null (Coalescent) and GMYC (mixed Yule Coalescent) priors; the value used for the likelihood ratio test (LR) and its associated probability (*p*), corresponding to a comparison between the single threshold and the null model, and the comparison between the multiple threshold and the single threshold models. The estimated number of Maximum Likelihood entities or “phylogenetic species” and the confidence interval (CI) around these values are reported. Statistically significant values are emphasised in bold. ML entities correspond to clades labelled in Figure 2.

## Discussion

The use of multiple molecular data sources enables the comparison of the evolutionary history of each gene and potentially more accurate estimation of the evolutionary history of the focal organisms (e.g. [58]). In the case of *B*. *harrisoni*, the combination of sequence data from four genes identified which genes are useful for identifying lineages with this level of divergence (e.g. *COI* and *PEPCK*) and which genes should be avoided in future studies (e.g. *16S* and *EF1α*). Furthermore, the combined sequence data resulted in a more resolved and well-supported *phylogenetic* hypothesis than when each of the individual datasets were analysed alone (Figures 2 and 3). This result is highlighted by the partitioned Bremer support values which show little incongruence (i.e. absence of negative and positive Bremer support values on a particular node) and, when the Bremer values are combined, provide moderate to strong support for the relationships of the major lineages (Figure 2), suggesting that single-gene datasets should be supplemented wherever possible [59,60]. Thus, the results presented here provide substantiate the approach of previous studies of the genus *Baetis*[15,16] and suggest means of refining them.

### Gene tree incongruence

The results produced from the *16S* gene were incongruent (but not significantly so) with the *COI* and nuclear (*PEPCK*) results, due to the recovery of a paraphyletic clade (SA East + MAL) (Table 2, Figure 3a). Possible explanations for incongruence include a lack of appropriately informative sites, substitution saturation, introgression, paralogy and/or incomplete lineage sorting. The *16S* gene is second to *COI* in its informative value (Table 1), although the data suffer from higher levels of homoplasy relative to the *COI* data (Table 1). Plots of transitions and transversions against genetic distance (not illustrated) showed that the *16S* data are not saturated. The mitochondrion is inherited as a single unit, thus the incongruence between the *16S* and *COI* data cannot be due to introgression, but may be due to incomplete lineage sorting of the *16S* gene.

The results produced from the *EF1α* gene were significantly incongruent with both the mitochondrial (*COI* and *16S*) and nuclear (*PEPCK*) results. Possible explanations for the observed pattern are limited to introgression and/or incomplete lineage sorting [61,62]. Although *EF1α* is known to occur in two copies in some insects [63,64], the lack of multiple PCR bands and the low between-clade divergence (0.03) estimated between the two clades would imply that only one copy has been amplified in this study. Although limited introgression is possible between a few samples in this study, it is not a viable explanation for the geographically distant samples, which were genetically indistinguishable (Figure 3d). Introgression in this case is also unlikely as there would have to have been multiple separate hybridisation events to result in the five distinct clades obtained from the *COI* and *PEPCK* data. Incomplete lineage sorting is the most likely explanation for the incongruence obtained between gene trees (Figure 3a-d), resulting from the random retention and extinction of alleles between species [61].

### Potential causes of lineage diversification

Although the dispersal of mayflies is thought to be limited to nearby water bodies because they are weak fliers with short adult lifespans [3], it has been shown that long-distance dispersal is more prevalent than previously thought [65]. The lack of within-clade isolation by distance apparent in this study (when excluding geographically isolated samples from Malawi and Zambia) would suggest high levels of gene flow within the three lineages in South Africa.

The role of catchments and their corresponding watersheds in structuring the genetic history of organisms in southern Africa is well documented (e.g. redfin barbs: [66,67]; atyid shrimps: [68,69]; and cicadas: [70,71]), although the impact of different catchments on invertebrates with aquatic stages is varied, with evidence both for (e.g. [72-74]) and against (e.g. [75,76]) a catchment effect, dependent primarily on the dispersal ability of the organism [77]. Although the SA East lineage is not found in the same catchments as the SA West lineage, the combination of the widespread distribution of the SA East and SA West lineages over multiple primary catchments and the range overlap between the SA Ced and SA West lineages would suggest that there is little-to-no effect of catchments influencing population structuring.

The tentative dating analyses are based solely on two estimates of the *COI* substitution rate and thus are to be interpreted with caution, but they provide plausible first estimates for the group. The estimated time to most recent common ancestor (TMRCA) for each clade is not markedly affected by the tree prior (Tables 3 &4; Additional file 2: Figure S1), but the between-clade divergence estimates depend on the choice of tree prior with a Yule prior favouring younger estimates (Tables 3 &4; Additional file 2: Figure S1). In this case the combination of Bayes Factor support for the Yule prior and the proposed status of each clade as recently diverged but distinct phylogenetic species support the use of the between-species Yule prior over the within-species Coalescent prior and the more complex between-species with extinction Birth-Death prior.

Between clade divergence estimates under the Yule prior suggest that the major cladogenic events within the group took place during the Pliocene to mid-Miocene (Tables 3 &4; Additional file 2: Figure S1), a pattern in common with previous studies in the region, focussing on aquatic or semi-aquatic freshwater invertebrates [78-82] and fish [67], strongly suggesting that common processes (outlined below) may have been responsible.

The TMRCA estimated within each lineage ranged from the youngest (MAL: 0.5–0.7 mya) to the oldest (SA East: 1.5–1.7 mya) when using the faster *COI* substitution rate, suggesting population fragmentation within the Plio-Pleistocene. Within the Plio-Pleistocene, aridity and seasonality in southern Africa increased with the intensification of the Benguela current and the formation of the winter rainfall zone [83,84] and global climate fluctuations in response to Milankovitch oscillations [85] which resulted in glacial cycles throughout the Pleistocene. These glacial cycles, and the associated aridity linked to glacial periods [86], probably resulted in the repeated fragmentation and bottlenecking of *Baetis* populations within southern Africa and have been previously cited as a population isolating mechanism within the region [71,87].

The lineages sampled in this study correspond to environmentally identified (pH-based) and summer versus winter rainfall variation. The SA Ced and SA West lineages comprise samples from streams that flow through acidic Table Mountain Sandstone geologies whereas the SA East, MAL and ZAM lineages comprise samples from streams with an alkaline pH. The difference in the pH range of the rivers is primarily a result of the local geology [14,25], so river pH is unlikely to have changed within the timeframe (Mio-Pliocene) required to result in the isolation of lineages. The response of many aquatic invertebrates to pH is well studied and it is known that the pH of a river affects its community composition [88,89]. Thus the lineages discussed in this study are each likely to have a restricted pH tolerance and this lineage-specific pH tolerance is the most likely mechanism for the continued genetic isolation between the parapatric SA West and SA East lineages of *B*. *harrisoni*. The Krom River (SA East, Figure 1, see Additional file 1: Table S1) and Elands River (SA West, Figure 1, see Additional file 1: Table S1) are approximately 30 km apart, yet the shift in geological composition results in a drastic change in pH over the short distance. The Elands River flows through areas of Table Mountain Sandstone (resulting in poorly-buffered acid waters) and the Krom River flows through Bokkeveld (mostly shale and sandstone composition which results in well-buffered neutral-to-alkaline waters) [90]. This mechanism requires experimental verification and cannot be invoked to explain the continued isolation between the sympatric SA West and SA Ced lineages.

### Taxonomic status of clades

Although the South African material used in this study has been well sampled (Figure 1), the GMYC results must be interpreted with caution as there are large sampling gaps between South Africa and Malawi / Zambia and the GMYC model is sensitive to sampling strategies, which may result in artificial clustering due to a lack of intermediate haplotypes. Within South Africa the lineages are found both in sympatry (Figure 1: SA Ced and SA West) and parapatry (Figure 1: SA West and SA East) and are estimated to share a common ancestor at least 2.4 mya (Tables 3 &4), suggesting five species under the phylogenetic species concept. The individual GMYC analyses resulted in the recognition of between 5 and 7 maximum likelihood (ML) entities, with the combined data favouring six ML entities. In analyses based on the *COI*, *16S* and the combined data, the SA East lineage was divided into two ML entities. As these two ML entities were not well supported monophyletic clades in all three (*COI*, *16S* and *PEPCK*) datasets, we have chosen a more conservative estimate of five monophyletic, well supported clades, corresponding to five phylogenetic species, here highlighted as: SA Ced, SA West, SA East, ZAM and MAL (Figure 2).

Furthermore the between-clade genetic distances estimated here are comparable to those found between distinct species both in this study (i.e. outgroup vs. ingroup) and in previous studies of *Baetis* species [1,15,16], providing strong evidence for the recognition of five species corresponding to each of the lineages mentioned using the phylogenetic species concept. Although subtle morphological variations have been observed in *B*. *harrisoni*, these findings require a thorough morphological investigation to assess whether there are diagnostic morphological characters and observable physiological adaptations to acid or alkaline pH conditions which are consistent and congruent with the molecular lineages.

The Afrotropical region is undoubtedly under-collected and material collected is often insufficiently studied. This may explain the low diversity of African *Baetis* species despite the high generic diversity in the Afrotropical Baetidae [91]. Further sampling within Africa is needed to determine the geographical extent of each of the lineages within *B*. *harrisoni sensu lato* and to assess the diversity of the other *Baetis* species in the region (e.g. *B*. *parvulus* Crass, *B*. *monikae* Kopelke, *B*. *permultus* Kopelke and *B*. *pseudogemellus* Soldán).

## Conclusions

In conclusion, consistent with studies of the Holarctic branch of the genus *Baetis*[15,16], molecular markers have succeeded in revealing genetic variation that is indicative of cryptic species in southern African samples of *B*. *harrisoni*. Furthermore *COI* is cross-validated by the nuclear marker *PEPCK* as suitable for recognising these cryptic taxa. The *16S* gene shows indications of reduced phylogenetic information for the between-lineage relationships in this group while *EF1α* shows indications of incomplete lineage sorting, and is not recommended for identification, a conclusion which might be extended to other molecular studies of *Baetis* species (e.g. [15,16]). The tentative dating analyses indicate that historical and geographical processes within the mid-Miocene and Pliocene and continued isolation in response to water pH underlie the diversity in the *B*. *harrisoni* clade. Careful examination of morphology in the light of the clades recognised by this molecular study is warranted, and will aid in the resolution of the nomenclatural problems associated with this group. Finally these results have highlighted the need for accurate taxonomy in widely-used indicator species.

## Abbreviations

Th. *COI*: *Cytochrome c oxidase I*; *16S*: *Small subunit ribosomal 16S rDNA*; *EF1α*:*Elongation factor 1 alpha*; *PEPCK*: *Phosphoenolpyruvate carboxykinase*; GMYC: General mixed yule-coalescent; IBD: Isolation By Distance; AMGS: Albany Museum, Grahamstown; MP: Maximum parsimony; BI: Bayesian Inference; TBR: Tree bisection and reconnection; PBS: Partitioned bremer support; MY: Million Years; UPGMA: Unweighted pair group method with arithmetic mean; UCLN: Uncorrelated Log-Normal; MCC: Maximum clade credibility; ntax: Number of specimens with sequence data; # Var: Number of variable; # Pi: Parsimony informative; % Pi: Percent parsimony informative; Bp: Total number of base pairs; # trees: Number of trees retained; score: Tree length; CI: Consistence index; RI: Retention index; TMRCA: Time to most recent common ancestor; LR: Likelihood ratio test; SA Ced: Samples collected from the Cederberg mountains and the Breede River district, which is a winter rainfall region; SA West: The entire winter rainfall region of South Africa, Cederberg inclusive; SA East: The eastern summer rainfall region of southern Africa, corresponding to samples from South Africa; MAL: Malawi; ZAM: Zambia.

## Competing interests

The authors declare that they have no competing interests.

## Authors’ contributions

LLPdC collected specimens, acquired the molecular sequences genetic, aligned sequences, drafted the manuscript and edited the final version. BWP conceived the phylogenetic analyses, and assisted with sequence alignment, interpretation of data and revision of the manuscript. HMB-J conceived the study, and provided specimens from outlying areas; HMB-J and MHV designed the study, coordinated and supervised the research group, and assisted the drafting and revision of the manuscript; MHV also acquired specimens and interpreted data. FCdM contributed material from Cederberg, Western and Eastern Cape rivers, and contributed ecological insights. NPB assisted with laboratory work and interpreted results. All authors contributed to manuscript writing and have read and approved the final manuscript.

## Supplementary Material

Additonal file 1 **Table S1.** GenBank Accession numbers, samples and localities. Clade labels correspond to Figures 2 and 3. (‘-’ indicates failure to amplify; * = sequence obtained from GenBank; EC = Eastern Cape; KZN = KwaZulu-Natal; LIM = Limpopo; MPU = Mpumalanga; NW = North West; RSA = South Africa; WC = Western Cape). Click here for file

Additonal file 2 **Figure S1.** Plot of the mean time to most recent common ancestor (TMRCA). Estimated using BEAST under differing tree priors and substitution rates. (**A**) mean COI substitution rate of 3.5% per MY; (**B**) mean COI substitution rate of 1.5% per MY. Error bars correspond to the estimated 95% HPD. Dashed lines denote approximate boundaries of geologic epochs: Oli. Oligocene; Mio. Miocene; Pli. Pliocene; Ple. Pleistocene. Node numbering corresponds to Figure 2. Click here for file
