# Supplementary material for: Cryptic variation in an ecological indicator organism: mitochondrial and nuclear DNA sequence data confirm distinct lineages of Baetis harrisoni Barnard (Ephemeroptera: Baetidae) in southern Africa
Source: BMC Evol Biol. 2012 Feb 29;12:26. doi: 10.1186/1471-2148-12-26 (PMC3523013; doi:10.1186/1471-2148-12-26)
Supplement: Additonal file 1 — Table S1. GenBank Accession numbers, samples and localities. Clade labels correspond to Figures 2 and 3. (‘-’ indicates failure to amplify; * = sequence obtained from GenBank; EC = Eastern Cape; KZN = KwaZulu-Natal; LIM = Limpopo; MPU = Mpumalanga; NW = North West; RSA = South Africa; WC = Western Cape). [file 1471-2148-12-26-S1.pdf]

Table S1: Samples and localities used in this study. Clade labels correspond to Figures 2 and 3. ('-' indicates failure to amplify; * = sequence obtained from GenBank; EC = Eastern Cape; KZN = KwaZulu-Natal; LIM = Limpopo; MPU = Mpumalanga; NW = North West; RSA = South Africa; WC = Western Cape).

Clade	Locality	AlbanyMuseum Cat. No.	Latitude / Longitude	Specimen Extraction Code	GenBank Accession Number			
					PEPCK	COI	16S	EF1α
OUT	VersoixSauverny, Switzerland	-	46°18'41"N / 06°07'13"E	llp 048	HM637081	HM636958	HM637016	HM637040
				llp 049	-	HM636959	HM637017	-
SA Ced	SuurvleiRiver, WC, RSA	CED 18B	32°37'44"S / 19°10'55"E	llp 032	-	HM636946	HM637001	-
	WitteRiver, WC, RSA	WCR 385A	33°34'18"S / 19°08'19"E	llp 022	HM637060	HM636939	HM636993	-
				llp 023	HM637061	-	HM636994	-
				llp 024	HM637062	HM636940	HM636995	HM637036
	WaterkloofRiver, WC, RSA	WCR 386A	32°32'07"S / 19°01'49"E	llp 072	HM637099	-	-	-
				llp 073	HM637100	HM636979	-	-
				llp 074	HM637101	-	-	-
SA West	BloukransRiver, WC, RSA	WCR 387A	33°57'19"S / 23°38'20"E	llp 027	HM637063	HM636941	HM636996	HM637037
				llp 028	HM637064	HM636942	HM636997	HM637038
				llp 053	-	HM636963	HM637020	HM637042
	ElandsRiver, WC, RSA	TSR92H	34°01'03"S / 24°03'39"E	llp 059	HM637088	HM636969	HM637026	HM637048
				llp 060	HM637089	HM636970	HM637027	-
				llp 061	-	HM636971	-	-
		TSR94A	34°01'03"S / 24°03'39"E	llp 062	HM637090	HM636972	HM637028	HM637049
				llp 063	HM637091	HM636973	-	-
				llp 064	HM637092	HM636974	-	-

Clade	Locality	AlbanyMuseum Cat. No.	Latitude / Longitude	Specimen Extraction Code	GenBank Accession Number			
					PEPCK	COI	16S	EF1α
	KaaimansRiver, WC, RSA	WCR 388A	33°58'19"S / 22°32'52"E	llp 029	HM637065	HM636943	HM636998	HM637039
				llp 030	-	HM636944	HM636999	-
				llp 054	HM637084	HM636964	HM637021	HM637043
	RondegatRiver, WC, RSA	CED 64D	32°22'24"S / 19°05'37"E	llp 034	HM637068	-	HM637002	-
				llp 035	HM637069	HM636947	HM637003	-
				llp 036	HM637070	HM636948	HM637004	-
	StormsRiver, WC, RSA	TSR21	33°59'19"S / 23°55'09"E	llp 013	HM637055	-	-	HM637033
				llp 014	HM637056	HM636933	HM636985	-
				llp 015	-	HM636934	HM636986	HM637034
	SuurvleiRiver, WC, RSA	CED 18B	32°37'44"S / 19°10'55"E	llp 031	HM637066	HM636945	HM637000	-
				llp 033	HM637067	-	-	-
SA East	GamtoosRiver, EC, RSA	GEN 1968A	33°49'10"S / 24°51'39"E	llp 010	HM637053	HM636931	HM636983	HM637032
				llp 011	HM637054	-	HM636984	-
				llp 012	-	HM636932	-	-
		GEN 1969B	33°50'07"S / 24°52'43"E	llp 069	HM637096	-	-	-
				llp 070	HM637097	HM636978	-	-
				llp 071	HM637098	-	-	-
	GreatFishRiver, EC, RSA	ECR 736A	33°04'29"S / 26°47'21"E	llp 050	-	HM636960	HM637018	HM637041
				llp 051	HM637082	HM636961	HM637019	-
				llp 052	HM637083	HM636962	-	-
	IxopoRiver, KZN, RSA	GEN 1965A	30°07'10"S / 30°10'59"E	llp 046	HM637079	HM636956	HM637014	-

Clade	Locality	AlbanyMuseum Cat. No.	Latitude / Longitude	Specimen Extraction Code	GenBank Accession Number			
					PEPCK	COI	16S	EF1α
				llp 047	HM637080	HM636957	HM637015	-
				llp 058	-	HM636968	HM637025	HM637047
	Kaaloog se Loop River, NW, RSA	GEN 1966A	25°46'37"S / 26°26'00"E	llp 043	-	-	HM637011	-
				llp 044	HM637077	HM636955	HM637012	-
				llp 045	HM637078	-	HM637013	-
	KarkloofRiver, KZN, RSA	GEN 1973A	29°28'12"S / 30°14'23"E	llp 039	HM637073	HM636951	HM637007	-
				llp 040	HM637074	HM636952	HM637008	-
				llp 056	HM637086	HM636966	HM637023	HM637045
	BloukransRiver, EC, RSA	GEN 1970A	33°19'29"S / 26°37'54"E	llp 037	HM637071	HM636949	HM637005	-
				llp 038	HM637072	HM636950	HM637006	-
				llp 055	HM637085	HM636965	HM637022	HM637044
	KromRiver, EC, RSA	GEN 1972A	33°57'21"S / 24°20'56"E	llp 065	HM637093	HM636975	-	-
				llp 066	HM637094	-	-	-
				llp 067	HM637095	HM636976	-	-
				llp 068	-	HM636977	-	-
	NgotwaneRiver, NW, RSA	GEN 1967A	25°27'19"S / 25°51'14"E	llp 041	HM637075	HM636953	HM637009	-
				llp 042	HM637076	HM636954	HM637010	-
				llp 057	HM637087	HM636967	HM637024	HM637046
	VaalRiver, MPU, RSA	-	26°00'06"S / 30°27'02"E	-	-	-	AJ971743*	-
	WitRiver, EC, RSA	GEN 1971B	33°21'01"S / 25°41'20"E	llp 004	HM637050	-	HM636980	HM637029
				llp 005	HM637051	HM636930	HM636981	HM637030
				llp 006	HM637052	-	HM636982	HM637031

Clade	Locality	AlbanyMuseum Cat. No.	Latitude / Longitude	Specimen Extraction Code	GenBank Accession Number			
					PEPCK	COI	16S	EF1α
MAL	Muswanga River, Malawi	CAW 403A	10°02'02"S / 33°42'22"E	llp 016	HM637057	-	HM636987	-
				llp 017	HM637058	HM636935	HM636988	HM637035
				llp 018	HM637059	-	HM636989	-
ZAM	Mahebe River, Zambia	CAW 402A	12°20'36"S / 25°54'28"E	llp 019	-	HM636936	HM636990	-
				llp 020	-	HM636937	HM636991	-
				llp 021	-	HM636938	HM636992	-
