# Supplementary material for: Cryptic variation in an ecological indicator organism: mitochondrial and nuclear DNA sequence data confirm distinct lineages of Baetis harrisoni Barnard (Ephemeroptera: Baetidae) in southern Africa
Source: BMC Evol Biol. 2012 Feb 29;12:26. doi: 10.1186/1471-2148-12-26 (PMC3523013; doi:10.1186/1471-2148-12-26)
Supplement: Additonal file 2 — Figure S1. Plot of the mean time to most recent common ancestor (TMRCA). Estimated using BEAST under differing tree priors and substitution rates. (A) mean COI substitution rate of 3.5% per MY; (B) mean COI substitution rate of 1.5% per MY. Error bars correspond to the estimated 95% HPD. Dashed lines denote approximate boundaries of geologic epochs: Oli. Oligocene; Mio. Miocene; Pli. Pliocene; Ple. Pleistocene. Node numbering corresponds to Figure 2. [file 1471-2148-12-26-S2.pdf]

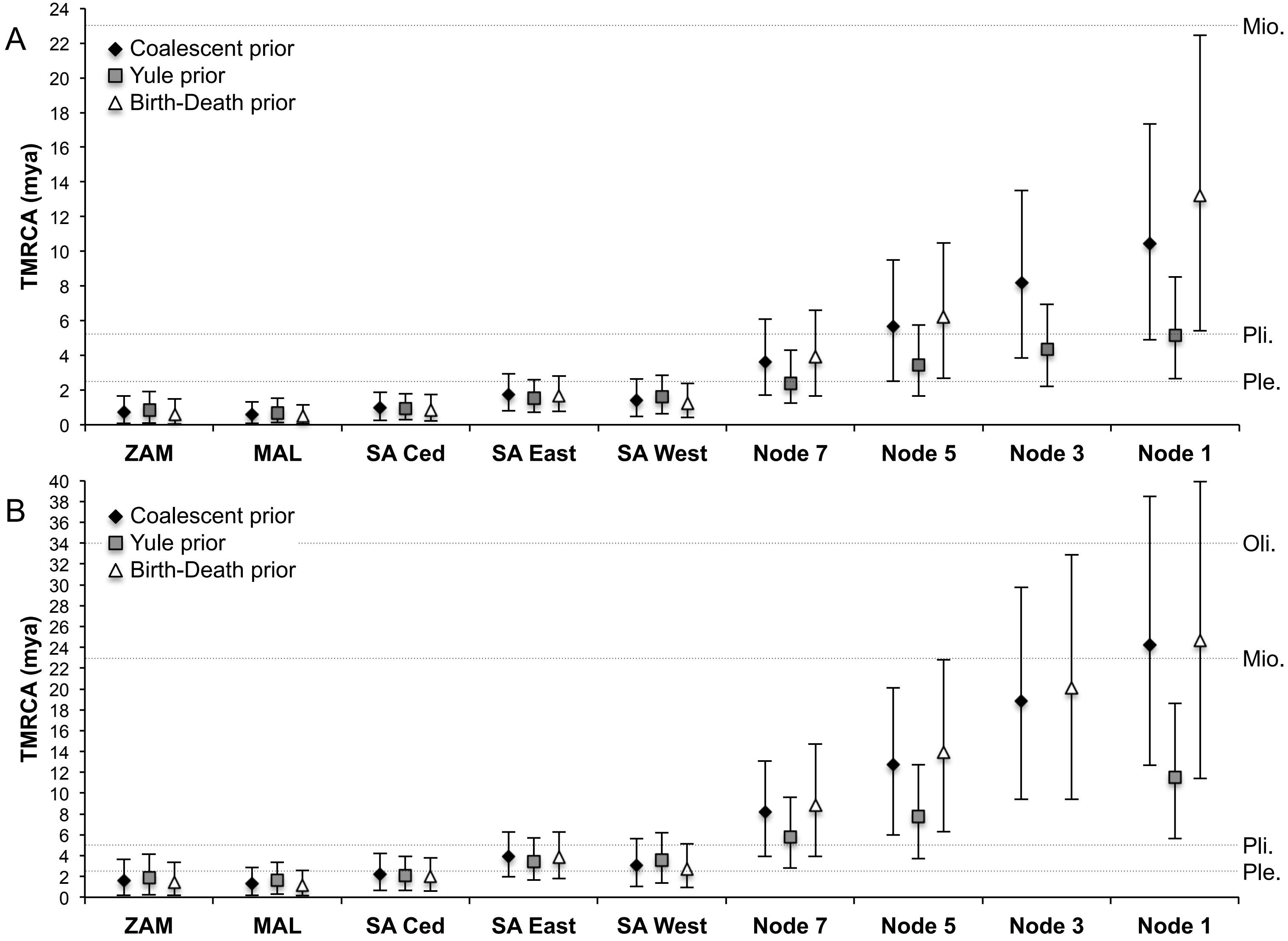

Figure S1. Plot of the mean time to most recent common ancestor (TMRCA) estimated using BEAST under differing tree priors and substitution rates. (A) mean *COI* substitution rate of 3.5% per MY; (B) mean *COI* substitution rate of 1.5% per MY. Error bars correspond to the estimated 95% HPD. Dashed lines denote approximate boundaries of geologic epochs: Oli. Oligocene; Mio. Miocene; Pli. Pliocene; Ple. Pleistocene. Node numbering corresponds to Figure 3.
